# Structural Covariance Networks in Children with Autism or ADHD

**DOI:** 10.1093/cercor/bhx135

**Published:** 2017-06-13

**Authors:** R. A. I. Bethlehem, R. Romero-Garcia, E. Mak, E. T. Bullmore, S. Baron-Cohen

**Affiliations:** 1 Autism Research Centre, Department of Psychiatry, University of Cambridge, Cambridge CB2 8AH, UK; 2 Department of Psychiatry, University of Cambridge, Cambridge CB2 0SZ, UK; 3 Cambridgeshire and Peterborough NHS Foundation Trust, Huntingdon PE29 3RJ, UK; 4 MRC/Wellcome Trust Behavioural and Clinical Neuroscience Institute, University of Cambridge, Cambridge CB2 3EB, UK; 5 Immuno-psychiatry, Immuno-Inflammation Therapeutic Area Unit, GlaxoSmithKline R&D, Stevenage SG1 2NY, UK; 6 CLASS Clinic, Cambridgeshire and Peterborough NHS Foundation Trust, Cambridge CB21 5EF, UK

**Keywords:** ADHD, autism, cortical thickness, graph theory, structural covariance

## Abstract

**Background:**

While autism and attention-deficit/hyperactivity disorder (ADHD) are considered distinct conditions from a diagnostic perspective, clinically they share some phenotypic features and have high comorbidity. Regardless, most studies have focused on only one condition, with considerable heterogeneity in their results. Taking a dual-condition approach might help elucidate shared and distinct neural characteristics.

**Method:**

Graph theory was used to analyse topological properties of structural covariance networks across both conditions and relative to a neurotypical (NT; *n* = 87) group using data from the ABIDE (autism; *n* = 62) and ADHD-200 datasets (ADHD; *n* = 69). Regional cortical thickness was used to construct the structural covariance networks. This was analysed in a theoretical framework examining potential differences in long and short-range connectivity, with a specific focus on relation between central graph measures and cortical thickness.

**Results:**

We found convergence between autism and ADHD, where both conditions show an overall decrease in CT covariance with increased Euclidean distance between centroids compared with a NT population. The 2 conditions also show divergence. Namely, there is less modular overlap between the 2 conditions than there is between each condition and the NT group. The ADHD group also showed reduced cortical thickness and lower degree in hub regions than the autism group. Lastly, the ADHD group also showed reduced wiring costs compared with the autism groups.

**Conclusions:**

Our results indicate a need for taking an integrated approach when considering highly comorbid conditions such as autism and ADHD. Furthermore, autism and ADHD both showed alterations in the relation between inter-regional covariance and centroid distance, where both groups show a steeper decline in covariance as a function of distance. The 2 groups also diverge on modular organization, cortical thickness of hub regions and wiring cost of the covariance network. Thus, on some network features the groups are distinct, yet on others there is convergence.

## Introduction

Autism spectrum conditions (henceforth autism) are characterized by deficits in social communication alongside unusually restricted interests and repetitive behaviors, difficulties adjusting to unexpected change, and sensory hypersensitivity (American Psychiatric Association 2013). Despite a large body of research to understand its underlying neurobiology (Loth et al. 2015), no distinct set of biomarkers for autism has yet been established. With respect to the neuroimaging literature and specifically network connectivity, several authors have suggested potential differences in brain organization in autism compared with neurotypical (NT) control groups. There is, however, debate about whether autism is characterized by neural over- or underconnectivity (Brock et al. 2002; Rubenstein and Merzenich 2003; Belmonte et al. 2004; Just et al. 2004; Courchesne and Pierce 2005). A traditional hypothesis is that people with autism suffer from atypical connectivity (Courchesne and Pierce 2005; Cherkassky et al. 2006; Just et al. 2007; Assaf et al. 2010). Specifically, there is a tendency for autism to be associated with excess local or short-range connectivity, relating to enhanced local processing. This is thought to be accompanied by decreased global or long-range connectivity, relating to impaired integration as manifested in “weak central coherence.” Thus, a prominent theory of neural connectivity in autism is of global under- and local overconnectivity (Belmonte et al. 2004; Vissers et al. 2012). Other, more recent theories have pointed towards more network dependent levels of dysconnectivity. Zielinski et al. (2012) reported a connectivity reduction in salience network and posterior regions of the default mode network (DMN), whereas frontal DMN regions were overconnected. This notion of network dependent alterations was recently confirmed by a large structural covariance study in the ABIDE dataset (Long et al. 2016). Interestingly, Long and colleagues also show how this network dependency seems to change with age. Lastly, regional covariance alterations in autism have also been demonstrated to persist in white matter microstructure (Dean et al. 2016). Dean and colleagues show an overall decreased coherence in individuals with autism that might suggest a broader pattern of dysconnectivity.

Attention-deficit/hyperactivity disorder (ADHD) on the other hand is characterized by a triad of symptoms: hyperactivity, impulsive behavior, and inattentiveness (American Psychiatric Association 2013). Studies using connectivity analyses have attempted to shed light on its underlying neurobiology and have found both decreased and increased functional connectivity in specific networks (Tomasi and Volkow 2012), altered connectivity in the DMN (Fair et al. 2010) and differences in cross-network interactions (Cai et al. 2015). These effects might be smaller than literature suggests (Mostert et al. 2016).

Autism and ADHD show high comorbidity and phenotypic overlap (Rommelse et al. 2010, 2011; Leitner 2014), and are both also potentially marked by differences in connectivity. There have even been suggestions that these connectivity differences lie on a similar dimension of local and global connectivity imbalances (Kern et al. 2015). In addition, both conditions have been associated with alterations in cortical development (Shaw et al. 2007; Hardan et al. 2009) that could in turn give rise to differences in the topological organization of brain networks. In the present study, we aimed to identify distinct as well as overlapping patterns of brain organization that might shed a light on the underlying architecture of both conditions, giving rise to divergent yet related findings using structural covariance analyses.

Structural covariance analysis involves covarying interindividual differences (i.e., coordinated variations in grey matter morphology) in neural anatomy across groups (Alexander-Bloch et al. 2013a; Evans 2013) and is emerging as an efficient approach for assessing structural brain organization. A key assumption underlying this methodology is that morphological correlations are related to axonal connectivity between brain regions, with shared trophic, genetic, and neurodevelopmental influences (Alexander-Bloch et al. 2013a). Thus, structural covariance network analysis is not the same as analysis of functional connectivity or structural networks obtained with diffusion imaging, yet it has shown moderately strong overlap with both (Gong et al. 2012; Alexander-Bloch et al. 2013a). In addition, structural covariance networks are highly heritable (Schmitt et al. 2009) and follow a pattern of coordinated maturation (Zielinski et al. 2010; Raznahan et al. 2011; Alexander-Bloch et al. 2013a). With respect to neurodevelopmental conditions, structural covariance networks might provide a way to investigate potential differences in brain network development. Differences between NT individuals and individuals with a developmental condition are likely the result of divergent developmental trajectories in coordinated development of different brain networks. The advantage of structural covariance analysis is that it focuses on this coordinated structure of the entire brain as opposed to focusing on a specific structure. In addition, structural data on which these networks are based is widely available, analysis is less computationally intensive and arguably less sensitive to noise, compared with functional imaging.

Previous investigations of structural covariance in autism have shown regional or nodal decrease in centrality, particularly in key regions subserving social and sensorimotor processing, compared with NT individuals (Balardin et al. 2015). Furthermore, speech and language impairments in autism have been associated with differences in structural covariance properties (Sharda et al. 2014). Studies of functional connectivity networks in autism are more abundant (Vissers et al. 2012). In ADHD structural covariance analyses have been extremely scarce. A study that specifically investigated structural covariance in drug-naïve adolescent males found that grey matter volume covariance was significantly reduced between multiple brain regions including insula and right hippocampus, and between the orbitofrontal cortices (OFC) and bilateral caudate (Li et al. 2015). Similar to the autism literature, studies that have explored functional connectivity differences in ADHD are more abundant (Konrad and Eickhoff 2010).

While autism and ADHD are considered distinct conditions from a diagnostic perspective, clinically they share some common phenotypic features (such as social difficulties, atypical attentional patterns, and executive dysfunction) and have high comorbidity (Rommelse et al. 2010, 2011; Leitner 2014). DSM-5 (American Psychiatric Association 2013) now allows comorbid diagnosis of autism and ADHD, acknowledging the common co-occurrence of these conditions. Regardless, most studies to date have focused on each condition separately, with considerable heterogeneity in results. Taking a dual-condition approach might help elucidate shared and distinct neural characteristics. Our proposal for a dual-condition approach is supported by a recent review that found both distinct as well as overlapping neural characteristics between autism and ADHD (Dougherty et al. 2015). There is also increasing interest in the clinical and research communities to investigate autism and ADHD along a continuum of atypical neural connectivity (Kern et al. 2015).

In the present study, we used the graph theoretical framework to analyse properties of structural covariance networks across autism and ADHD, relative to an age and gender matched NT group. One study has taken a similar approach using resting-state fMRI and diffusion weighted tractography and reported marked connectivity differences between network hubs, indicating a disruption in rich-club topology. Specifically, Ray et al. (2014) report a decrease in connectivity within the rich-club but increased connectivity outside the rich-club in ADHD. The autism group showed an opposite pattern of increased connectivity within rich-club connectivity. These findings may fit with the idea of increased local connectivity in autism (i.e., increased within rich-club connectivity), with ADHD showing the opposite pattern. Yet, these findings could also mediate increased strength in long-range connections within the rich-club. In the present study we aimed to further investigate the relation between distance and connectivity by looking at group-wise cortical thickness covariance as a function of Euclidean distance. In addition, we investigate potential overlap in modular and hub organization as assessed by structural covariance network analyses.

## Methods

### Image Processing and Quality Control

Structural T1-weighted MPRAGE images were collected from 2 publically available datasets: ABIDE (http://fcon_1000.projects.nitrc.org/indi/abide/) and ADHD-200 (http://fcon_1000.projects.nitrc.org/indi/adhd200/). From these datasets, 3 diagnostic groups (autism, ADHD, and NT individuals) of males between the ages of 8 and 12 years old were selected. The initial sample consisted of 348 eligible individuals. The structural T1-MPRAGE data were preprocessed using Freesurfer *v5.3* to estimate regional cortical thickness. Cortical reconstructions were checked by 3 experienced independent researchers. Images were included in the analyses only when a consensus on the data quality was reached (see Supplementary Information for more details on data selection). The cortical thickness maps were automatically parcellated into 308 equally sized cortical regions of 500 mm^2^ that were constrained by the anatomical boundaries defined in the Desikan–Killiany atlas (Desikan et al. 2006; Romero-garcia et al. 2012). The backtracking algorithm grows subparcels by placing seeds at random peripheral locations of the standard atlas regions and joining them up until a standard predetermined subparcel size is reached (Romero-garcia et al. 2012). It does this reiteratively (i.e., it restarts at new random positions if it fails to cover an entire atlas region) until the entire atlas region is covered. Individual parcellation templates were created by warping this standard template containing 308 cortical regions to each individual MPRAGE image in native space. A key advantage of warping of the segmentation map to the native space relates to the attenuation of possible distortions from warping images to a standard space that is normally needed for group comparisons. Lastly, average cortical thickness was extracted for each of the 308 cortical regions in each individual participant.

As a secondary post hoc step in quality control, individuals that had an average variability in cortical thickness of more than 2 standard deviations away from the group mean were removed from further analysis. After quality control and matching on age and IQ, our final sample consisted of 218 participants: ADHD (*n *= 69, age = 9.99 ± 1.17, IQ = 107.95 ± 14.18), autism (*n *= 62 age = 10.07 ± 1.11, IQ = 108.86 ± 16.94) and NT (*n* = 87, age = 10.04 ± 1.13, IQ = 110.89 ± 10.39). See Supplementary Information Figure S1 for an overview and Table S1 for details on scanner site and matching procedure. Scanner site was regressed out from raw cortical thickness estimates across groups. To aid interpretation of the cortical thickness estimates, the residuals from this regression where added to the sample mean. Group-wise structural covariance matrices were then computed by taking the inter-regional Pearson correlation of these parcel-wise cortical thickness estimation. This was done within each group to create group-wise structural covariance matrices.

## Data Analysis

### Group Differences of Distance Effects in CT Covariance

To determine potential group effects on the CT covariance for short and long-range associations, we investigated the linear slope differences in the relationship between correlation strength and Euclidean distance between nodal centroids. Consequently, one-way analysis of covariance (ANCOVA) were performed with the diagnosis group as a factor and Euclidean inter-regional distance as a covariate. For significant group effects, post hoc paired *t*-tests were used to identify which slopes are significantly different from each other.

### Graphs

To construct adjacency matrices for graph analyses, the minimal spanning tree (van Wijk et al. 2010) was used as the threshold starting point for building covariance networks at a representative density of 10%. The density of a network relates to the fraction of edges present in the network compared with the maximum possible number of edges. Graph analyses were performed across densities and between-group differences were compared using nonparametric permutation tests on paired group comparisons (1000 permutations). Thus, permuted networks were constructed by permuting the underlying cortical thickness estimates for each group comparison and constructing adjacency matrices for each. In view of the large number of comparisons across the 308 nodes, differences in local measures were subjected to a false discover rate (FDR) nonlinear multiple comparison correction with alpha set at <0.025 to allow simultaneous correction for two-tailed testing (Benjamini and Hochberg 1995).

### Degree, Cortical Thickness and Wiring Cost Analysis

Nodal degree reflects the number of edges connecting each node. Nodes with the highest degree of the network are defined as hubs. The present study considered a wide range of degree thresholds to reduce bias related to the choice of an arbitrary set of hubs (ranging from 0% to 100% of the nodes). Thus, group differences in degree and CT of the hubs of the networks were evaluated for each degree threshold. To decrease the noise effect, we calculated the cumulative degree distribution as $P\left(k\right)=\sum_{k^{\prime }\geq k}p(k^{\prime })$.

Inter-regional distance ($d_{\mathrm{ij}}$) between 2 nodes *i* and *j* was estimated as the Euclidean distance between the centroids, $d_{\mathrm{ij}}=\sqrt{{(x_{i}-x_{j})}^{2}+{(y_{i}-y_{j})}^{2}+{(z-z_{j})}^{2}}$, where *x*, *y*, and *z* represents the coordinates of the centroid of each region in MNI space. The mean connection distance or wiring cost $(W_{c})$ of a network was computed as, $W_{c}=(\sum_{i,j}{\mathrm{net}}_{\mathrm{ij}}*d_{\mathrm{ij}})/N$, where net*(i,j)* is equal to one if regions *i* and *j* are connected, 0 otherwise, and *N* is the total number of connections of the network.

### Modular Agreement

Modular agreement was evaluated by quantifying the proportion of pairs of regions that were classified within the same module in community partitions (using iterating Louvain clustering to obtain modular partitions) associated with different diagnostic groups. Thus, 2 groups will show high modular agreement if network modules mainly include the same set of brain regions in both groups. As modular agreement is highly affected by intrinsic trivial characteristics of the modular partition, *z*-scores were used as a measure of how over- or under-represented a given metric was compared with random community partitions. In order to test against appropriately designed surrogate data, statistical significance was assessed against a null distribution built from metric values computed in 1000 random communities generated by preserving the number of modules, size of the modules, spatial contiguity and hemispheric symmetry of the real community partition. The 95th quantile of the resulting distribution was used as a statistical threshold to retain or reject the null hypothesis of no significant modular agreement between diagnostic groups. Moreover, differences in modular agreement between pairs of groups were statistically tested using a similar procedure. Indices of modular agreement of each pair of groups were subtracted and compared with the differences of modular agreement derived from the 1000 random communities in each pair of groups. Similarly, the 95th quantile of the resulting distribution was used as a statistical threshold to retain or reject the null hypothesis of no modular agreement differences between pairs of diagnostic groups. Significant results were corrected for multiple comparisons using FDR (Benjamini and Hochberg 1995).

## Results

### Distance Covariance Topology

**Figure 1.**
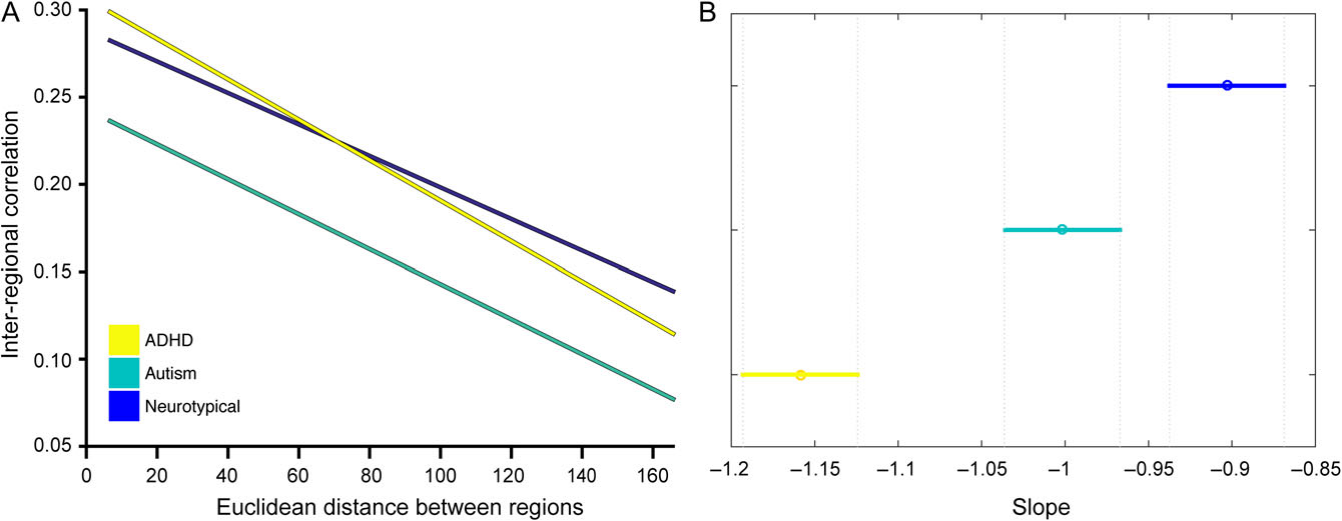
Inter-regional correlation strength as a function of Euclidean distance. (*A*) The inter-regional correlation over the entire distance range. (*B*) The mean slope for each group and the 95% confidence interval of the mean slope.

In all groups the group-wise correlation strength decreased with increased anatomical distance. Results from the analysis of variance show a main effect of group *F*_2,141828_ = 2192.76, *P* < 0.0001. Post hoc analyses indicated that all 3 groups have a small but significantly different slope: ADHD < NT (*P* < 10^−15^), autism < NT (*P* < 0.005) and ADHD < autism (*P* < 10^−15^). Figure 1 shows the linear relation of the inter-regional correlation as a function of Euclidean distance and the mean and confidence intervals of the slope estimates. In the ADHD group, inter-regional correlation decreased the fastest whereas the NT group shows the smallest decreases. This result shows that both autism and ADHD have relatively weaker long-range covariance and stronger local covariance. Compared with the NT group both groups show a balance that more strongly favors short-range over long-range covariance.

### Degree

**Figure 2.**
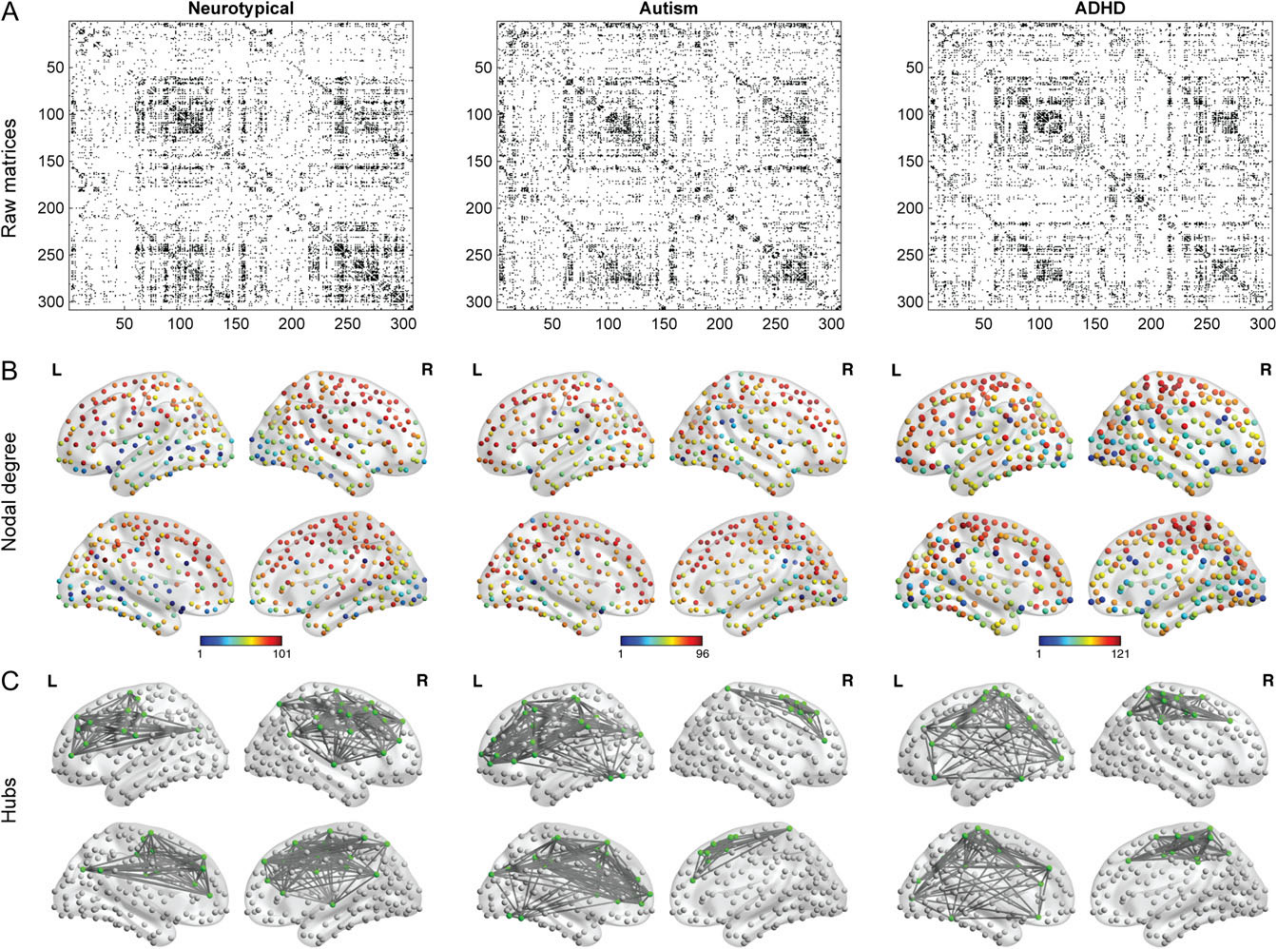
Overview of procedure and metrics. (*A*) The binary adjacency matrices for the 3 groups thresholded at 10% above the minimal spanning tree. Subsequent graph construction is based on these thresholded matrices. (*B*) The topological distribution of nodal degree at 10% density. (*C*) The networks with nodes that have the highest degree (top 10%).

**Figure 3.**
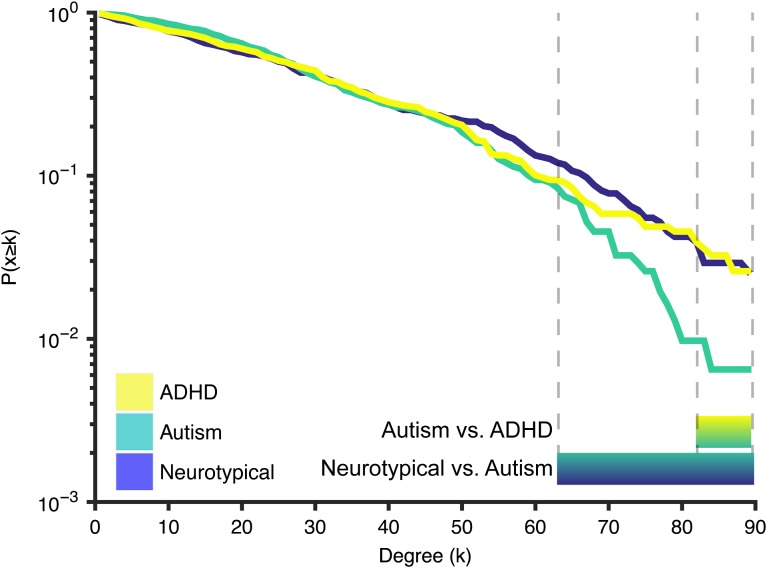
Cumulative degree distribution. Lines represent the proportion of nodes in the network with a degree higher than *k* (hubs) in each group. Bars below the figure represent the areas where there is a significant difference between the groups. Hubs of the autism group showed significantly lower degree compared with the ADHD group (*k*-range: 83–88) and compared with the neurotypical group (*k*-range: 64–89).

After constructing the covariance matrices (Fig. 2*A*), the degree of each node was computed (Fig. 2*B*) and the top 10% nodes with highest degree were retained as hubs for visualization (Fig. 2*C*). Most of the hubs were located within frontal and parietal cortices in the 3 groups. In contrast, nodes with lower degree were mainly placed in the occipital cortex. There were several nodes that showed degree differences between groups, but these were not consistent across degree densities. We did, however, observe marked differences between groups in the overall degree distribution. Figure 3 shows the cumulative degree distribution of each group. Interestingly, hubs of the autism group exhibited significantly lower degree than both NT (*P* < 0.025; for degree values from 83 to 88) and ADHD (*P* < 0.025; for degree values from 64 to 89). These difference were corrected for multiple comparisons for the range of higher degree nodes (FWE correction in the degree range from 50 to 90).

### Wiring Cost

**Figure 4.**
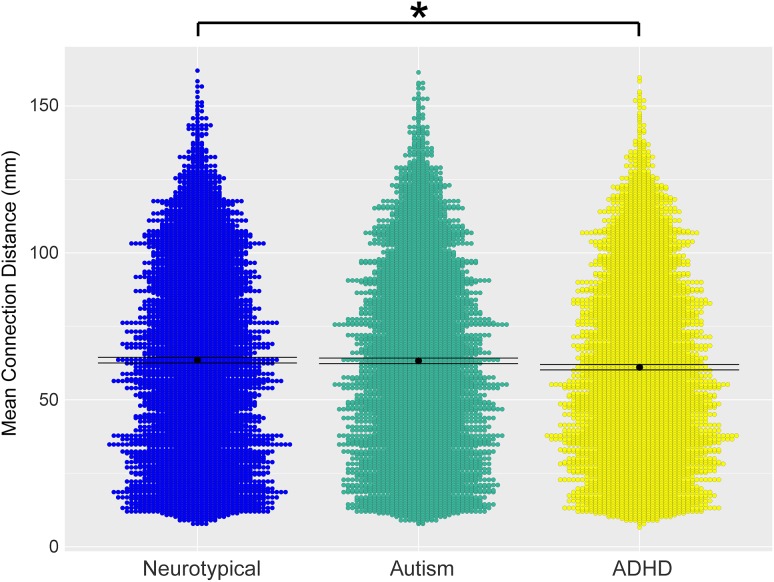
Violin representation of the mean inter-regional distance between connected regions in the 3 groups. The ADHD group has significantly lower connection distance compared with the neurotypical group. Mean is shown as a black dot with error bars representing 95% confidence intervals

In line with the group differences observed in the decay of cortical thickness correlation as a function of the inter-regional distance described above, the wiring cost analysis showed a significant decrease of the average distance between connected regions in the ADHD group compared with NT (Fig. 4; *P* < 0.008), revealing a reduction of long-range connections in the ADHD network.

### Cortical Thickness as a Function of Degree

**Figure 5.**
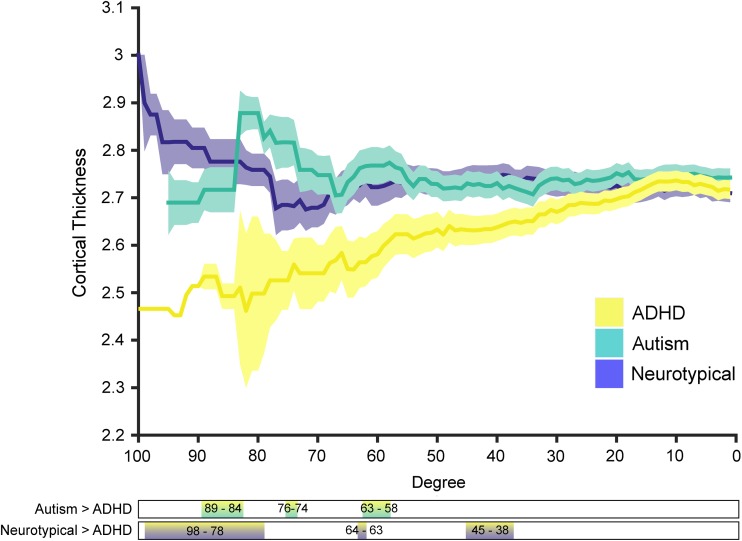
Cortical thickness as a function of degree, shaded areas indicate the standard deviation of the mean. Bars below the figure show the degree ranges where there is a significant difference between the respective groups.

Given that there were notable differences in degree distributions (i.e., hubs in the autism group had lower degree than the other groups; Fig. 2) we chose to analyze both the absolute degree distribution and take a percentile that was based on the group itself. Although the autism and NT group showed little difference in cortical thickness across the entire range of degrees with both methods, high-degree nodes had significantly reduced cortical thickness in the ADHD group (Fig. 5). This suggests that there might be increased synaptic pruning in these hub regions in the ADHD group.

### Modular Consistency and Clustering

**Figure 6.**
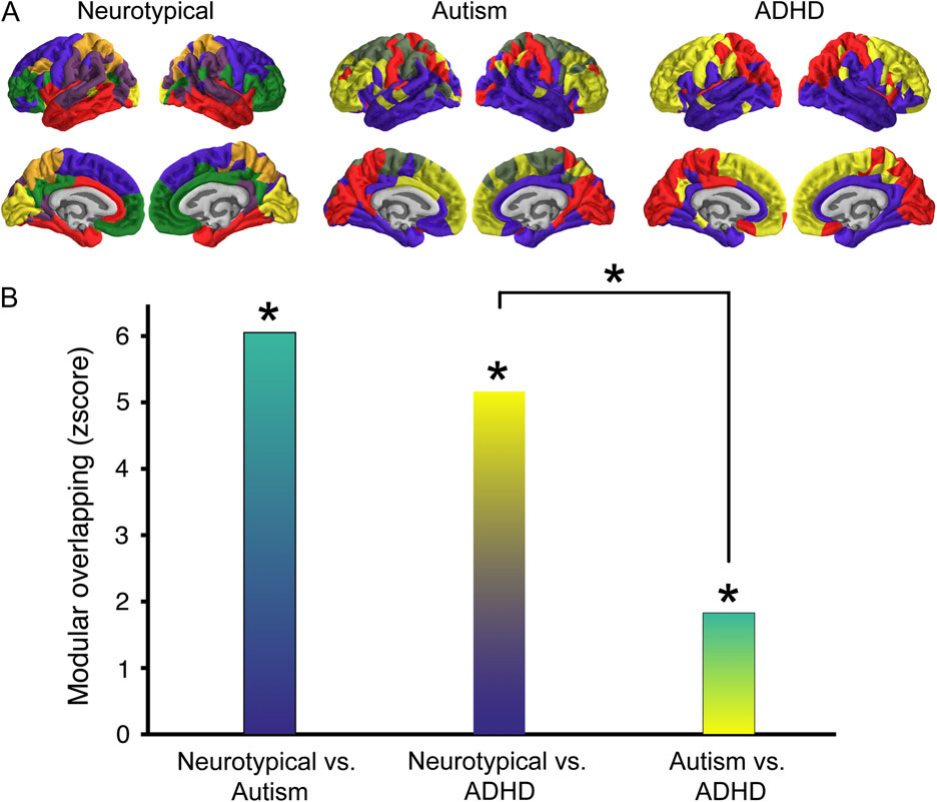
Similarities in community structure across groups. (*A*) The modular organization of the structural covariance network derived from each group. The colors show association of the region with a certain module. These colors are set for each group individually as not all groups have the same number of modules. (*B*) The *z*-transformed modular overlap for each group-wise comparison, color meshes are chosen to represent the group comparison. All overlap scores are significantly different from zero, indicating that nodes in one module are most likely part of the same module in both groups. Note that autism–ADHD overlap was reduced compared with the NT–ADHD overlap.

To investigate similarities in global topology, we further evaluated the modular overlap between the community structure of the 3 groups. The modular overlap between all group-wise comparisons were significantly higher than expected by chance (Fig. 6), suggesting that a global scale there were no marked differences in structural covariance community structure. However, the autism–ADHD group overlap was significantly lower than the NT–ADHD overlap (*P* < 10^−3^). There was also a small nonsignificant effect for the NT–ADHD overlap compared with NT-autism (*P* = 0.04). This indicates that although there were perhaps no massive topological differences in community structure, the autism and ADHD group differ more from one another than they do from the NT group (i.e., there was lower modular agreement between autism and ADHD then there was between the other groups).

Lastly, we have extended our main findings using covariance networks based on inter-regional correlation of the local gyrification index (LGI). Structural covariance network based on LGI also showed a significant reduction of the nodal degree in highly connected nodes in Autism compared with NT group (see Supplementary Information Figure S4). In line with the reduction of CT in the hubs of Autism and ADHD compared with NT groups described in Figure 5, we have found a reduction of LGI in ADHD compared with NT groups for highly connected nodes. Overall, these results suggest that both thickness and gyrification of high-degree nodes are particularly affected in these conditions.

## Discussion

Comparing autism and ADHD, our findings reveal a complex topology of convergent yet distinct patterns of brain network organization. At a global level of community structure all groups show a significant degree of overlap, however, the autism and ADHD group showed less similarity than they do compared with the NT group. The decay of cortical thickness correlation strength as a function of inter-regional distance was also markedly different for both clinical groups. Fitting with the idea of a local versus global connectivity difference in developmental conditions both the autism and ADHD group showed a pattern that diverges from the NT group. Yet, they do not appear to be in opposing direction. Both groups showed a significantly stronger decrease in correlation strength with increased distance relative to a control group.

These findings seem to suggest that in both conditions the topology favors short-range correlations over long-range correlations. This idea is prominent in autism literature, but less so in the ADHD literature. For example, Schaer and colleagues observed increased covariance in cortical folding in individuals with autism in short-range but not in long-range connections (Schaer et al. 2013). It will be interesting for future studies on different modalities such as resting-state or DTI imaging to see if potential connectivity differences follow a pattern similar to the present structural covariance properties. In addition, we found that the ADHD group had a marked decrease in cortical thickness in high-degree regions compared with the other 2 groups. A previous study showed that children with ADHD exhibited reduced CT in frontoparietal regions, but increased CT in occipital regions (Almeida Montes et al. 2013). In the present analysis cortical hubs were mainly located in frontoparietal networks, thus this finding fits with the idea of overall reduced CT in those areas. Interestingly, Almeida-Montes and colleagues also show that some of these difference increase with age. This would also fit with previous work showing some delay in cortical maturation of cerebrum and specifically prefrontal cortex in children with ADHD (Shaw et al. 2007).

A previous study indicated that wiring costs in autism might also fit in a model of increased local connectivity and decreased global connectivity in grey matter connections (Ecker et al. 2013b). Thus, we extended our local versus global analysis to include wiring cost characteristics. We found that the ADHD group showed significantly reduced wiring cost. This would be consistent with the notion of a network shift towards increased segregation (i.e., more local connections) at the expense of global integration. We did not find a significant difference in the wiring cost for the autism group. The present approach to assess wiring costs differs significantly from the one taken by Ecker et al. (2013b) (e.g., we use Euclidean distance between centroids of anatomically derived nodes compared with a measure of mean separation distance on the cortical sheet). It is possible that our approach might be too coarse to pick up wiring cost differences in the autism group. Our results do indicate a sharp reduction in the number of connections of the hubs regions in the autism network. Under-connected hubs could indicate a reduced capability of integrating information over the long-range and across modalities, something that has often been speculated to be the case in autism (Happé and Frith 2006). Again, future studies will have to show whether these patterns also emerge from connectomic data.

Since changes in structural covariance are postulated to be a result of a prolonged developmental process, our findings also provide emerging evidence for a systematic difference in the developmental trajectory/profile of brain organization between these groups. However, a recent large cross condition analysis of potential genetic relationship showed only moderate genetic overlap between autism and ADHD (Lee et al. 2013). Thus, the true underlying cause for these differences is likely more indirect and could emerge from long-term differences in functional connectivity. In relation to that, phenotypic overlap might perhaps also be sought in a more indirect causal relationship. Unfortunately the present data does not allow a detailed analysis of phenotypic or trait overlap (due to the lack of overlapping measures between the 2 datasets). Leitner (2014) lists a number of converging points in ADHD and autism etiology, perhaps most strikingly the difficulties with social interaction. Although the profile, and possibly the cause, of social difficulties likely differs between children with autism or ADHD, problems with social interaction are found in both (Leitner et al. 2014). Perhaps these have a concordant effects on brain networks as this is a critical element of brain development (Blakemore 2010).

Contrary to our predictions, and in contrast to a previous study (Ray et al. 2014) that used a different imaging modality, we did not find any significant differences in rich-club topology between any of the groups. The rich-club coefficient indicates that high-degree nodes are more likely to connect to other high-degree nodes (sometimes summarized as “the rich cling together”). Although the structural covariance networks were constructed from T1-MPRAGE data, we had expected to find overlap between the fMRI, DTI and our current results. It would be highly interesting to see how these differences develop further. Connectivity findings in adult autism and ADHD are notoriously heterogeneous (Konrad and Eickhoff 2010; Vissers et al. 2012), so some developmental neuroanatomical differences might gradually change with age. The present data was restricted to a very specific age group and developmental changes continue long after this time frame. It would be interesting to see whether the currently observed lack of differences in structural covariance topology propagate in the same direction. More research is needed to assess these potential longitudinal changes in this population.

Modular organization of the network of the 3 groups revealed no significant differences, but instead showed significant overlap. Therefore, network nodes belonging to one module in one group are likely to belong to the same module in the other group. Considered in the clinical context of overlapping phenotypes and high comorbidity, the present results strengthen the notion that these 2 conditions should not be studied in isolation. However, the 2 clinical groups (despite being significantly similar) show less modular similarity to one another than they do compared with a NT group. However, both groups also showed significant overlap with the NT group, suggesting that the neuroanatomical differences between the clinical and control groups operate on more fine-grained scales (such as might be observed in graph theoretical measures). This finding shows that when these groups are studied solely in contrast with a NT group no difference might be observed on this metric.

There are some caveats surrounding the current study. First, and in contrast to some studies, we used cortical thickness estimates to construct our structural covariance network, thereby excluding subcortical regions from network analysis. Separate analyses of subcortical volumetric and covariance differences for the present data are however included in the Supplementary materials. To be able to combine cortical and subcortical regions, some studies have used covariance of grey matter volume instead (Balardin et al. 2015b). However, grey matter volume relies on the relationship between 2 different morphometric parameters, cortical thickness and surface area. Cortical thickness and surface area are both highly heritable but are unrelated genetically (Panizzon et al. 2009), leading to different developmental trajectories across childhood and adolescence (Herting et al. 2015). The combination of at least 2 different sources of genetic and maturational influence into a unique descriptor of cortical volume may act as a confounding factor that hinders a clear interpretation in the context of cortical covariance based networks. This is particularly relevant in conditions such as autism and ADHD where differences in cortical thickness, cortical volume and surface area are highly heterogeneous (Wolosin et al. 2009; Ecker et al. 2013a).

In light of the recent proliferation in graph theoretical studies, several semantic caveats should also be clarified to facilitate cross-comparisons of findings. While we have adopted the term “structural covariance” to characterize the macroscale connectome, the same terminology has also been used to describe structural networks that are indicative of atrophy patterns in neurodegenerative conditions (Seeley et al. 2009). In the latter approach, the covariance networks are typically derived from voxel-wise seed-based correlations, the seed being defined as a focal site of atrophy as found using voxel-based morphometry. Distinct from these restricted patterns of pathology-related networks, other studies have derived whole-brain networks on the basis of pairwise correlations between the structural morphology (i.e., volume, thickness, gyrification) across brain regions (Lerch et al. 2006; Alexander-Bloch et al. 2013b; Mak et al. 2016). The second difference concerns the morphology of interest in deriving the structural networks. For instance, volume-based intensities are inherently limited by the geometric convergence of surface area and cortical thickness (Ashburner and Friston 2000), both of which may be underpinned by distinct genetic and developmental factors. In contrast, cortical thickness provides a physical property of the cortical mantle by explicitly modeling the boundaries between the white matter and pial surface (Fischl and Dale 2000). Despite the differences in the construction of the networks, both approaches are similar in that the networks are derived at the group-level, thereby precluding single-subject analyses and/or correlations against clinical data. The central tenet of both approaches similarly rests upon the assumption that strong correlations—particularly those that exceed an arbitrary threshold—reflect underlying connectivity between regions (Alexander-Bloch et al. 2013a).

Secondly, it is possible that in both publically available datasets, some participants might have been comorbid for the other condition (e.g., individuals in the ABIDE might have had comorbid ADHD, and vice versa). Although all individuals in these datasets were diagnosed under the DSM-IV criteria, which does not allow this type of comorbidity, without the availability of more detailed diagnostic data, comorbidity or general phenotypic overlap cannot be ruled out completely. In addition, sample size restrictions would not allow a further subdivision within the presentation type of the ADHD group, which could be an interesting avenue for future research. Yet the primary aim of this study was to investigate overlap between the 2 conditions. If the present results were due to the individuals that shared this comorbidity, this would still support a common underlying neural architecture. Nonetheless, future longitudinal studies need to disentangle this overlap more precisely and in relation to specific phenotypic overlap as well as the trajectory of topological changes over time.

In sum, we found convergence between autism and ADHD, where both conditions show stronger decrease in covariance with increased Euclidean distance between centroids compared with a NT population. The 2 conditions also show divergence. Namely, there is less modular overlap between the 2 conditions then there is between each condition and the NT group. The ADHD group also showed reduced cortical thickness and higher degree in hubs regions compared with the autism group. Lastly, the ADHD group also showed reduced wiring costs compared with the autism group. Future research investigating these patterns in functional and structural connectivity and relating findings to behavioral or phenotypic data will hopefully shed light on the convergent and divergent neural substrates of autism and ADHD. Our findings do support the notion that both developmental conditions involve a shift in network topology that might be characterized as favouring local over global patterns. Lastly, they highlight the value of taking an integrated approach across conditions.

## Supplementary Material

Supplementary Data
