# Supplementary material for: Structural Covariance Networks in Children with Autism or ADHD
Source: Cereb Cortex. 2017 Jun 13;27(8):4267–76. doi: 10.1093/cercor/bhx135 (PMC5903412; doi:10.1093/cercor/bhx135)
Supplement: Supplementary Data [file bethlehem_et_al_autism_adhd_structcov_si2_clean2.docx]

Supplementary Information

Structural covariance networks in children with autism or ADHD

Bethlehem^1,2*±^, R.A.I., Romero-Garcia^2*^, R. Mak^2^, E., Bullmore, E.T.^2,3,4,5^, & Baron-Cohen, S^1,6^.

1. Autism Research Centre, Department of Psychiatry, University of Cambridge, Cambridge, CB2 8AH, United Kingdom.
2. Department of Psychiatry, University of Cambridge, Cambridge, CB2 0SZ, UK.
3. Cambridgeshire and Peterborough NHS Foundation Trust, Huntingdon, PE29 3RJ, UK.
4. MRC/Wellcome Trust Behavioural and Clinical Neuroscience Institute, University of Cambridge, Cambridge, CB2 3EB, UK.
5. Academic Discovery Performance Unit, GlaxoSmithKline R&D, Stevenage SG1 2NY, UK.
6. CLASS Clinic, Cambridgeshire and Peterborough NHS Foundation Trust, United Kingdom

* Authors contributed equally

± Corresponding author:

Richard A.I. Bethlehem

Autism Research Centre,

Douglas House,

18b Trumpington Road,

CB2 8AH, Cambridge

UK

[*rb643@medschl.cam.ac.uk*](mailto:rb643@medschl.cam.ac.uk)

## Sampling, quality control and matching

All scans were visually inspected by two independent researchers and only when both researchers agreed were subjects included in subsequent analyses. Additionally, subjects that had a whole-brain variance in cortical thickness higher than 3 standard deviations from the sample mean were also removed from subsequent analysis. After the quality control, there were a few sites that only contained 2 or fewer subjects (MaxMun, Olin, Pitt, Stanford and Trinity), in order to minimize the effect of regressing out site, these sites were removed from subsequent analyses. Secondly, two sites had more than 10 subjects selectively from only one group (Washington University and Peking), as regressing out these sites would effectively also remove potential group effects these sites were removed from further analyses. This left a total of 218 subjects; ADHD (*n=69, age = 9.99* ±*1.17, IQ = 107.95* ±*14.18*), autism (*n=62 age=10.07* ±*1.11, IQ = 108.86* ±*16.94*) and NT (*n=87, age = 10.04* ±*1.13, IQ = 110.89* ±*10.39*). Within the ADHD group subdivisions could be made between presentation types, namely; ADHD-Combined (n=38), ADHD-Hyperactive/Impulsive (n =9) and ADHD-Inattentive (n=22)


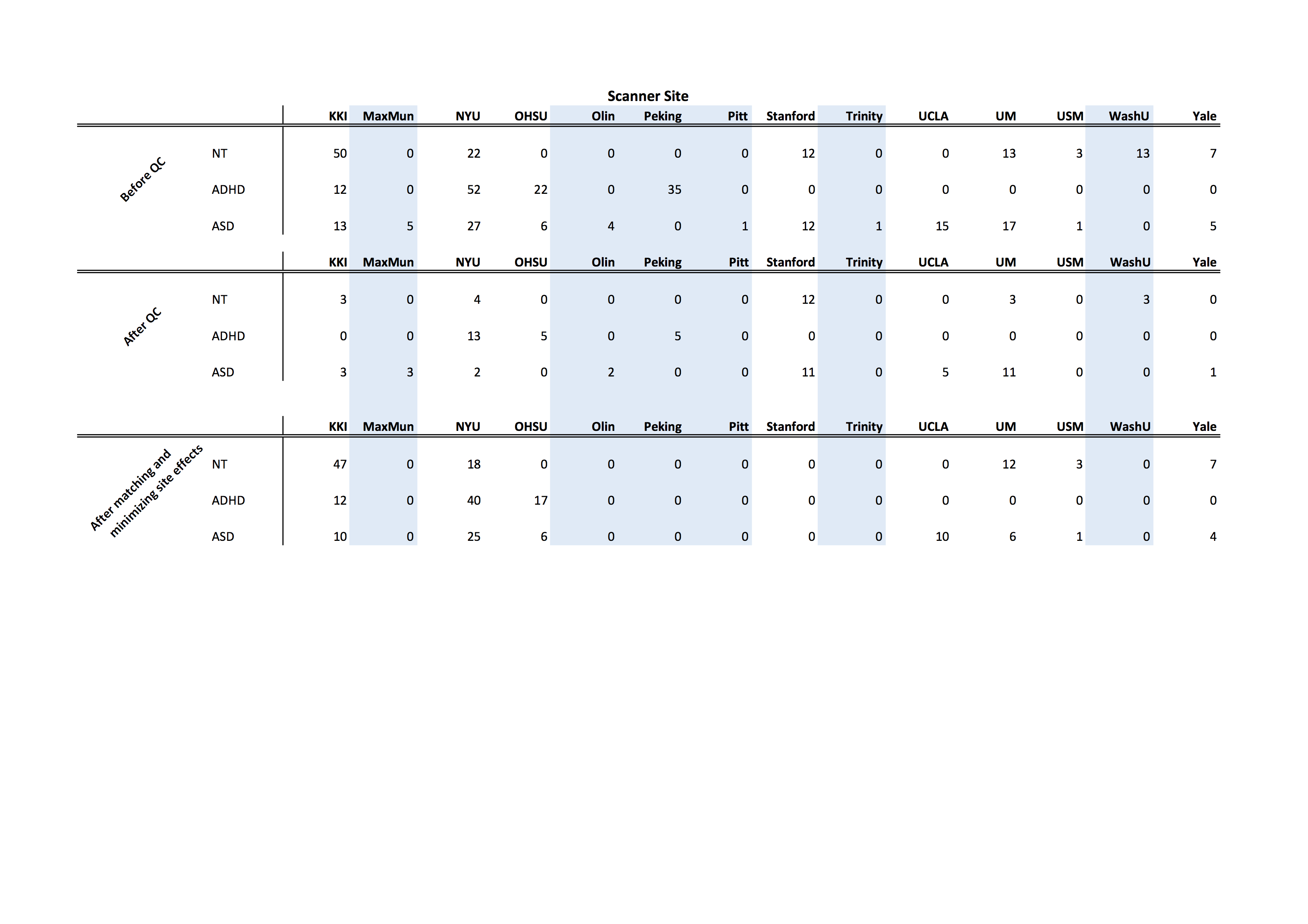


**Table S1:** Information on scanner site and matching

## Subcortical volumetric and covariance group differences

We found a significant volume difference between the Neurotypical and ADHD group in the left putamen, left amygdala and right amygdala (p-value <0.025, uncorrected; only the right amygdala survived FDR correction; Figure S2). We also found a volumetric difference between the ADHD and Autism groups in left amygdala and right amygdala, p-value <0.025, uncorrected; here only left amygdala survived FDR correction). The construction of a brain covariance network that included both cortical and subcortical structures was not suitable given that subcortical structures have a large variability of volume compared with the homogeneity of cortical regions with the same area as obtained from our parcellation scheme. However, we have explored the potential volume covariance of subcortical structures among groups (Figure S3). Here, we found that the autism group had, compared with neurotypical individuals, a significantly reduced covariance between right amygdala and left & right thalamus and between right amygdala and left Pallidum. Interestingly, the amygdala had a significantly reduced degree in the autism group compared to the control group. The ADHD group showed a significant correlation reduction between left & right cerebellum and left & right putamen, between left caudate and right thalamus and between left accumbens and right accumbens. Comparing the autism and ADHD groups revealed significant differences between left putamen and left & right cerebellum and between left pallidum and right amygdala. However, due to the large number of comparison these differences did not survived FDR corrections. The notion of altered subcortical and cortical processing fits with the idea of network alterations being dependent on the specific network (Zielinski et al., 2012). Specifically, reduced Involvement of amygdala and thalamic regions in the autism group might contribute to the underconnectivity of the salience network reported by Zalienski and collesgues (2012). Furthermore, although this can not be thoroughly assessed with the present approach (e.g. due the nature of the parcellation at the cortical level) it would seem reasonable to assume that these subcortical alterations in both volume and covariance could contribute to alterations in cortical-subcortical coupling, specifically of the amygdala and striatal regions (Eisenberg, Wallace, Kenworthy, Gotts, & Martin, 2015).

## Supplementary Figures


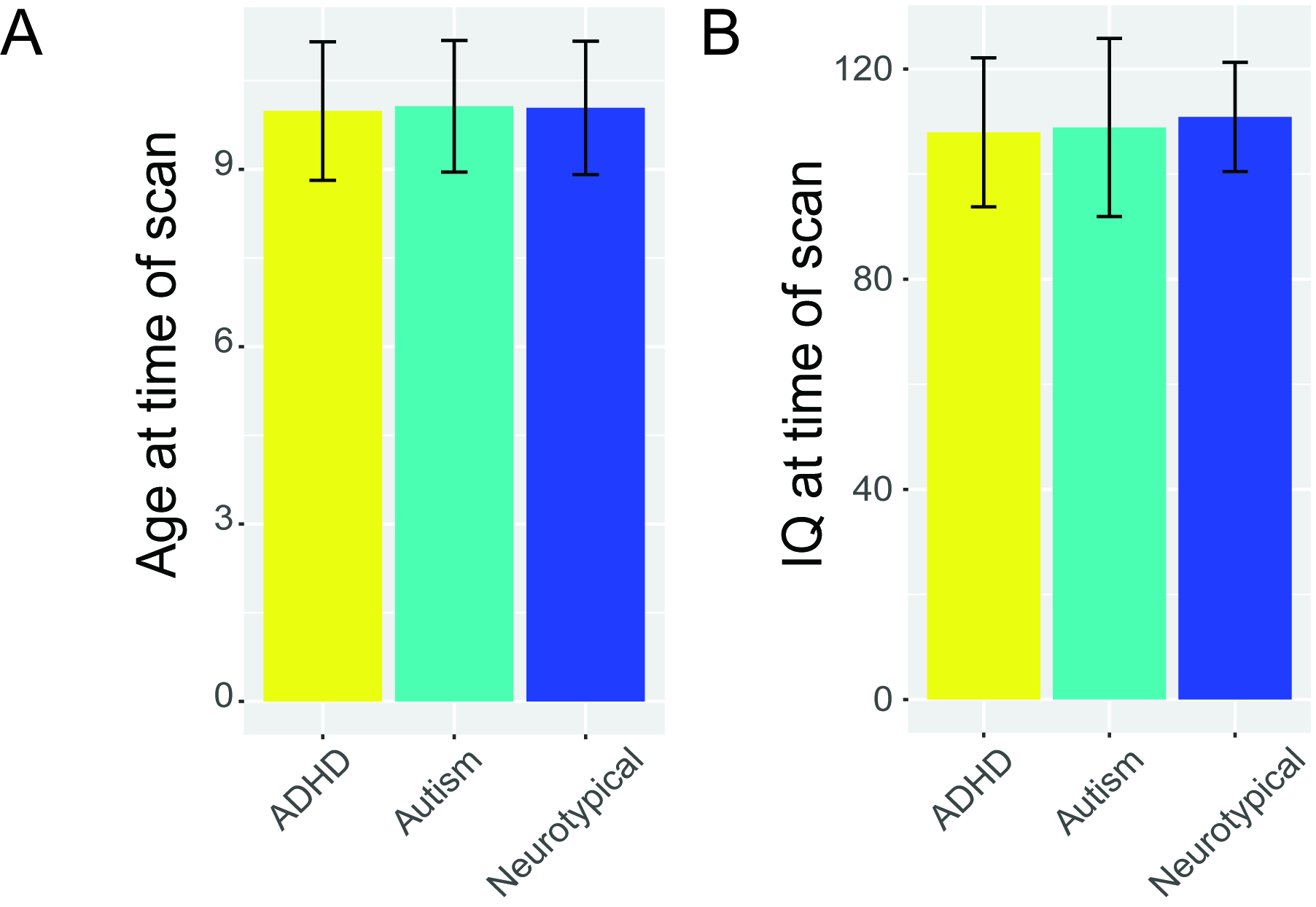


**Figure S1**: Age and IQ of all three matched groups. Panel A shows the mean age at time of scan, panel B shows mean IQ for each group. Error bars indicate standard deviation from the mean.


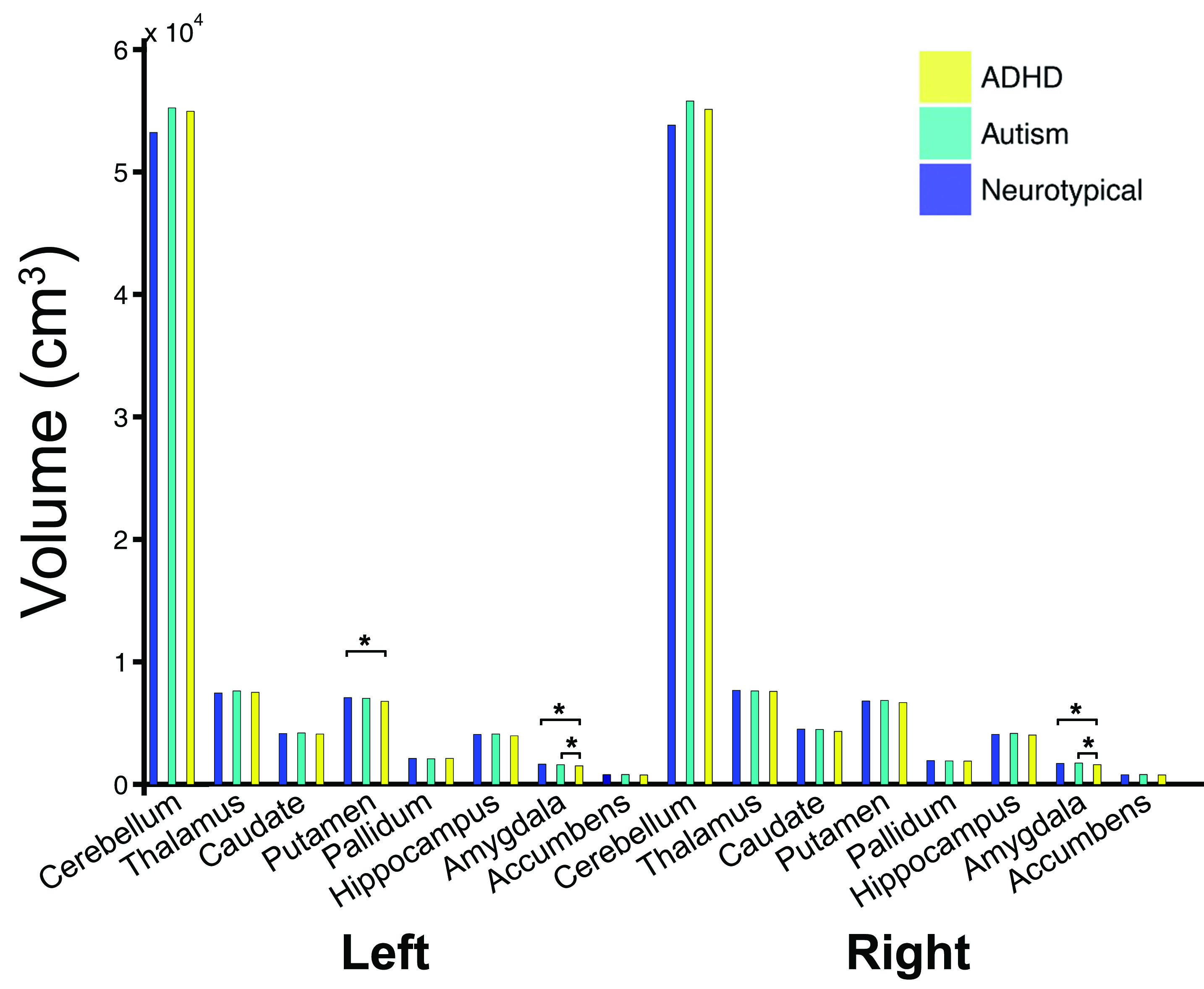


**Figure S2:** Volume of subcortical structures for Neurotypical, Autism and ADHD in the left and the right hemisphere. Asterisks denote significant difference between groups (p<0.025, uncorrected).


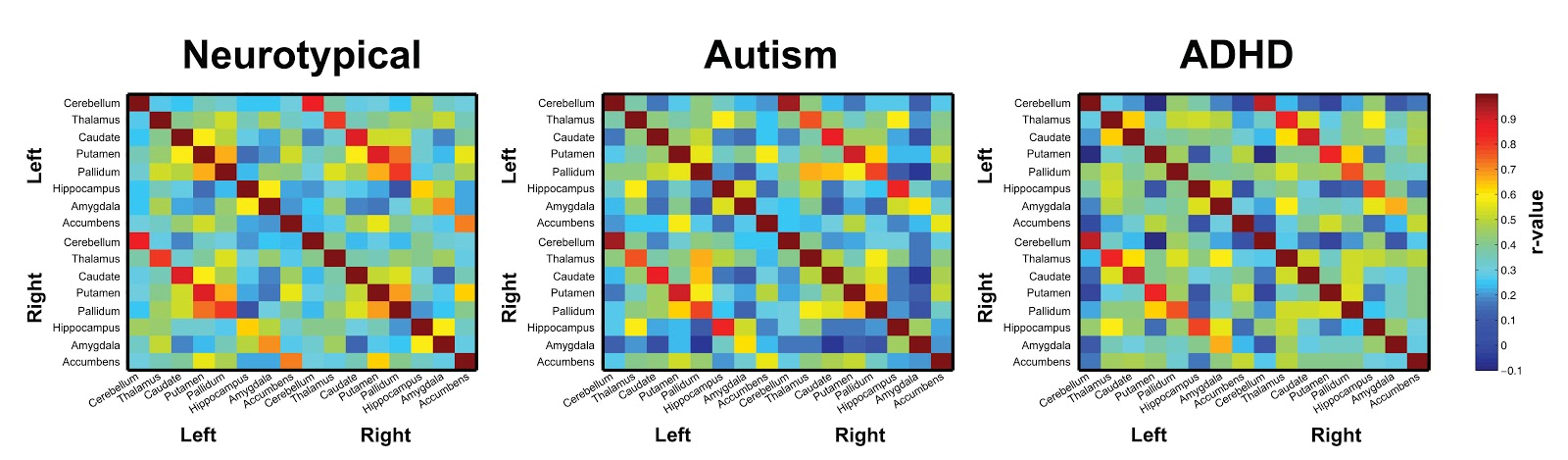


**Figure S3:** Subcortical covariance networks based on regional volume correlations.


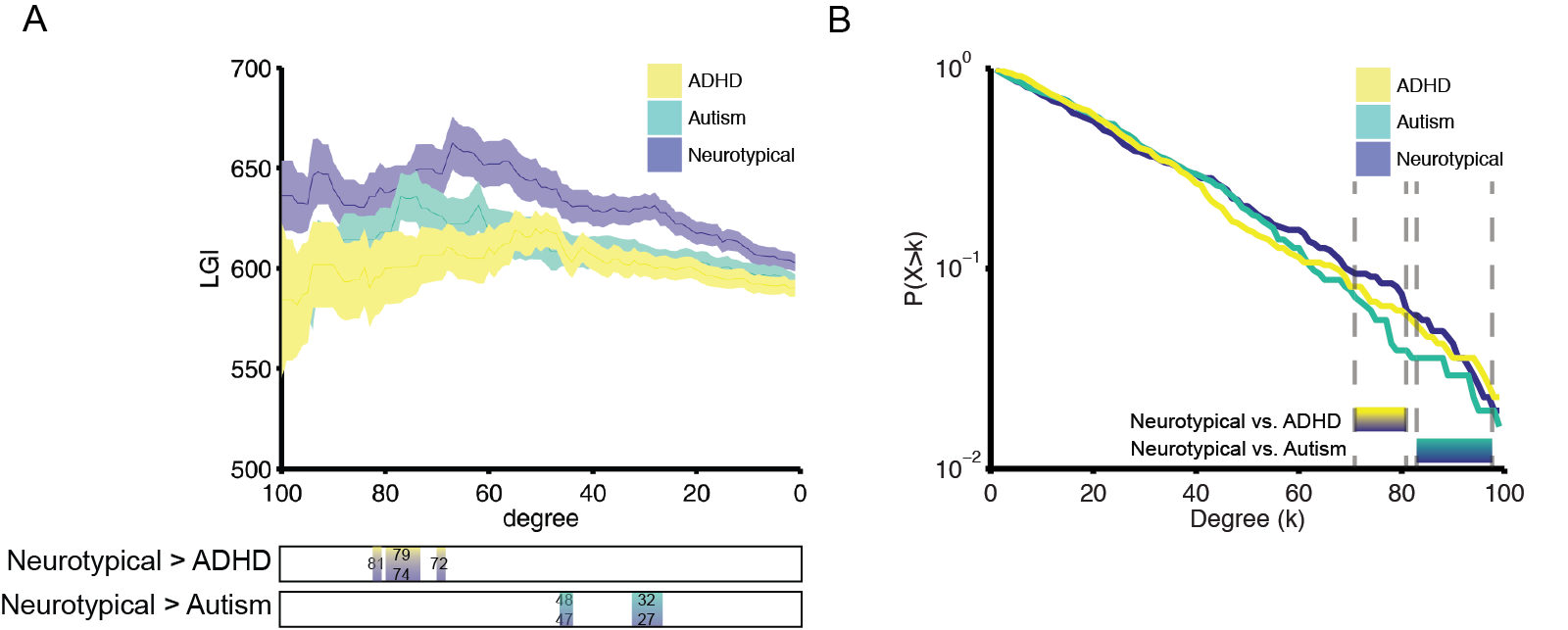


**Figure S4:** Local Gyrification Index (LGI) and degree distribution. Panel A shows LGI as a function of nodal degree. Bars below the figure show the degree ranges where there is a significant difference between the respective groups. Panel B shows the cumulative degree distribution of the covariance network based on LGI correlations**.** Lines represent the proportion of nodes in the network with a degree higher than k (hubs) in each group. Bars below the figure represent the areas where there is a significant difference between the groups.
